# Radionuclides and Radiation Indices of High Background Radiation Area in Chavara-Neendakara Placer Deposits (Kerala, India)

**DOI:** 10.1371/journal.pone.0050468

**Published:** 2012-11-21

**Authors:** Mary Thomas Derin, Perumal Vijayagopal, Balasubramaniam Venkatraman, Ramesh Chandra Chaubey, Anilkumar Gopinathan

**Affiliations:** 1 Division of Medical Biotechnology, School of Biosciences and Technology, VIT University, Vellore, India; 2 Radiological Safety Division, Indira Gandhi Center for Atomic Research, Kalpakkam, India; 3 Genetic Toxicology and Chromosome Studies Section, Cell Biology Division, Bhabha Atomic Research Centre, Mumbai, India; Kagoshima University Graduate School of Medical and Dental Sciences, Japan

## Abstract

The present paper describes a detailed study on the distribution of radionuclides along Chavara – Neendakara placer deposit, a high background radiation area (HBRA) along the Southwest coast of India (Kerala). Judged from our studies using HPGe gamma spectrometric detector, it becomes evident that Uranium (^238^U), Thorium (^232^Th) and Potassium (^40^K) are the major sources for radioactivity prevailing in the area. Our statistical analyses reveal the existence of a high positive correlation between ^238^U and ^232^Th, implicating that the levels of these elements are interdependent. Our SEM-EDAX analyses reveal that titanium (Ti) and zircon (Zr) are the major trace elements in the sand samples, followed by aluminum, copper, iron, ruthenium, magnesium, calcium, sulphur and lead. This is first of its kind report on the radiation hazard indices on this placer deposit. The average absorbed dose rates (9795 nGy h^−1^) computed from the present study is comparable with the top-ranking HBRAs in the world, thus offering the Chavara-Neendakara placer the second position, after Brazil; pertinently, this value is much higher than the World average. The perceptibly high absorbed gamma dose rates, entrained with the high annual external effective dose rates (AEED) and average annual gonadal dose equivalent (AGDE) values existing in this HBRA, encourage us to suggest for a candid assessment of the impact of the background radiation, if any, on the organisms that inhabit along this placer deposit. Future research could effectively address the issue of the possible impact of natural radiation on the biota inhabiting this HBRA.

## Introduction

Natural radiation is largely caused by the presence of primordial radionuclides and their decay products. Previous investigations reveal that the sources of radiation could vary from place to place but the dose rate generally falls between 80 and 150 nGy hr^−1^ world over [1], [2]. However, there are areas in some part of the world wherein the background radiation levels have been found to be abnormally high. Such areas are referred to as High Background Radiation Areas (HBRAs). Accordingly, the coastal regions of Espirito Santo and the Morro Do Forro in Brazil [3], [4] Yangjiang in China [5], [6] Southwest coast of India [7]–[10] Ramsar and Mahallat in Iran [11], [12] are identified as HBRAs. Monazite sands have been found to be the source of such high background radiation levels in certain parts of Brazil, China, Egypt and India [1], [3], [12] while in parts of Southwest France, uranium minerals form the source of natural radiation [13], and in Ramsar, the very high amounts of ^226^Ra and its decay products brought to the surface by hot springs [11], [12] have been found to be the source. In India, the occurrence of monazite sand bearing placer deposits, causing natural radiation along its long coastline, has been reported [1]. Ullal in Karnataka [14], Kalpakkam [15] in Tamilnadu, and coastal parts of Kerala state, and the Southwestern coast of India are known to be HBRAs [10]. Research in these places has generated considerable interest primarily due to geological reasons inasmuch as monazite, the rich source of radioactive uranium and thorium, becomes an important component in the sand from HBRAs [16]. Besides monazite, the beach placer deposits may contain zircon, ilmenite, rutile, and garnet. Further, the possible impact of natural radiation on the biota (including the humans) has been a matter of serious concern from societal and biological stand-points. Some of these areas have been under study for several years with a view to assess the risks and effects of long-term exposure to natural radiation, particularly with respect to the human inhabitants [11], [17], [18], [19]. However, the varying results on the natural radioactivity obtained by the previous investigations from the same locality [20], [21] have prompted us to undertake a study focusing on the Chavara – Neendakara placer deposits situated along the Southwest coast of India (Kerala), one of the very well known HBRAs in the world. Not only that the present paper provides us with an estimate of the radioactivity prevailing in Chavara – Neendakara, the authors have also assessed, for the first time, the various parameters of radioactive hazard indices (such as the absorbed gamma dose rates, the annual external effective dose rates and the annual gonad absorbable doses) from this HBRA. The types of trace elements in the soil sample have also been computed using EDAX, and are presented in this paper.

## Materials and Methods

### Description of the Study Area

**Figure 1:**
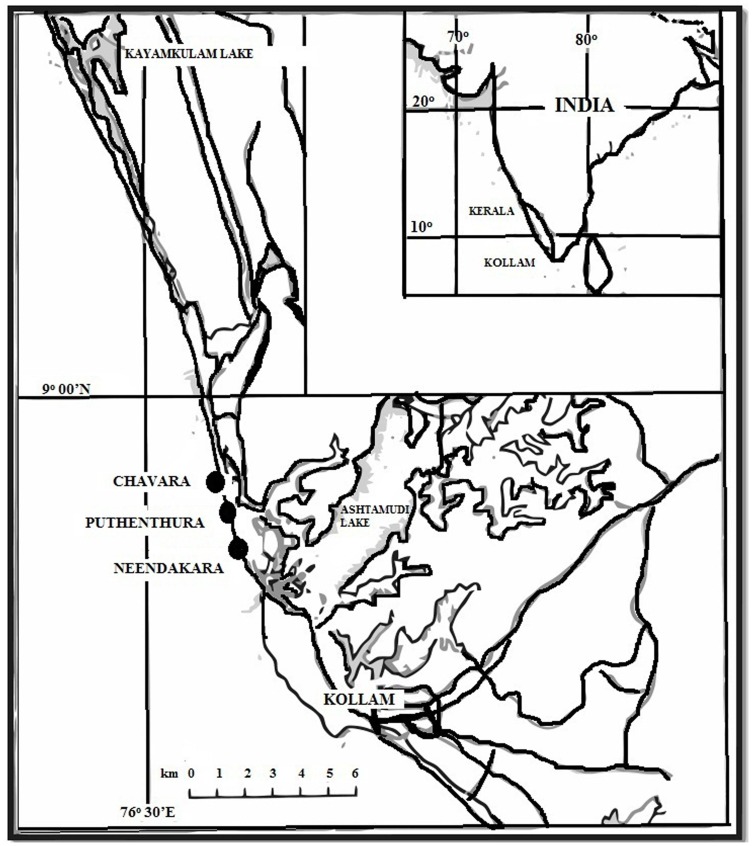
Map showing sample locations in the high background radiation area (HBRA) Chavara, Puthenthura and Neendakara in Kollam District, Kerala, India.

The study area includes two adjoining places such as Chavara (8°57.8′N 76°31.8′E) and Neendakara (8°56.8′N 76°32.1′E), along the Southwest coast of India (Kerala State), covering a coastal stretch of about 22 km, categorized as HBRA [10]. The study area is known as Chavara-Neendakara placer deposits as shown in Figure 1.

For the present study, 19 sampling locations that showed high activity (measurable from 1 meter above the ground level) read on a radiation survey meter were chosen. Sand samples were scooped out from a depth of 15–25 cm from each sampling points using a clean, pointed hand shovel [23]. About 1.5 kg of sand was collected from each location for analysis. The samples were stored in clean polythene bags for easy transportation to the laboratory. In the laboratory, the sand samples were dried in oven at 110°C for 12 hours to remove any moisture content. The processed sand samples were kept air tight in standard geometry plastic containers of 250 ml capacity. Alleppey (9°29′28.06′′N, 76°19′5.18′′E) and Chennai (13°3′18.55′′N, 80°17′4.18′′E) were identified as the Normal Background Radiation Areas (NBRAs), and were used as controls. About 1.5 kg of sand sample was collected from 10 sampling locations at a distance of 10 meter intervals from both the control areas for the present study. While collecting the samples, sufficient care was taken to ensure conformance to EML Procedures Manual [22].

No specific permits were required for the described field studies. The location is not privately - owned or protected in any way. The field studies did not involve endangered or protected species.

### Gamma Radiation Survey

Radiation levels were measured using a portable plastic scintillometer (PM1405, Polimaster) measuring micro-Sievert per hour (µSv h^−1^), with an accuracy of ±10, calibrated using different radiation sources (from Environmental Survey Laboratory, Kalpakkam, India).

### Radiometric Analysis

All the samples were subjected to gamma spectral analysis using high-resolution gamma spectrometry system consisting of coaxial p-type HPGe detector having 50% relative efficiency with respect to 7.62 cm × 7.62 cm NaI (Tl) detector. The energy resolution of HPGe detector, measured in terms of full-width at half maximum (FWHM) is 1.9 keV at 1332.5 keV of ^60^Co gamma energy at 25 cm from the top of the detector. Spectrum stabilized 8 k MCA (APTEC MCA) along with auto bias card were coupled with the HPGe detector. The detector was shielded with 7.5 cm lead to reduce the background contribution of the surroundings. Certified reference materials IAEA RGU, RGTh and RGK were used for energy and efficiency calibration of the system in the energy range from 46.53 to 2614.53 keV. The activity contents of the IAEA reference materials in 250 ml bottle geometry are known within ±3% accuracy. The samples sealed in radon-impermeable plastic containers, were stored for more than 30 days to bring ^222^Rn and its short-lived daughter products into equilibrium with ^226^Ra. The samples were then counted in the same source-to-detector geometry as used for the establishment of the efficiency calibration. The spectra were acquired for 20,000 sec and the photo peaks were evaluated using the APTEC MCA software. Gamma–ray photo peaks corresponding to 1.461MeV (^40^K), 1.764MeV (^214^Bi) and 2.615MeV (^208^Tl) were considered in arriving at the activity of ^40^K, ^226^Ra and ^232^Th in the samples. The detection limit of HPGe detector system for ^40^K, ^226^Ra and ^232^Th were 21.76 Bq kg^−1^, 3.39 Bq kg^−1^ and 6.64 Bq kg^−1^ respectively, for a counting time of 20,000 sec.

### Measurement of Radiation Hazard Indices

Radium equivalent activity (Ra_eq_), a widely used hazard index, is calculated by:
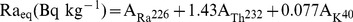
(1)


Where *A_Ra_* is the specific activity of ^226^Ra (which is the same as that of ^238^U) in Bq kg^−1^, *A_Th_* is the specific activity of ^232^Th in Bq kg^−1^, and *A_K_* is the specific activity of ^40^K in Bq kg^−1^.

The absorbed dose rates (D) due to gamma radiation in air at 1 meter above the ground surface, assuming uniform distribution of the naturally occurring radionuclides (^226^Ra, ^232^Th and ^40^K), has been calculated based on guidelines provided by UNSCEAR, 2000 [1]. This calculation was performed after assuming that the contributions from other naturally occurring radionuclides are insignificant. The value of D was calculated by:
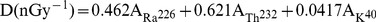
(2)


To estimate the annual external effective dose rates, the conversion coefficient from absorbed dose in air to effective dose (0.7 SvGy^−1^) and an outdoor occupancy factor (0.2) proposed by UNSCEAR 2000 [1], were used. Accordingly, the annual effective dose rate (mSv yr^−1^) (AEED) was calculated by:
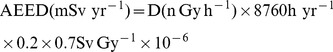
(3)


The annual gonadal dose equivalent (AGDE) due to the specific activities of ^226^Ra, ^232^Th and ^40^K was calculated using the formula proposed by Mamont-Ciesla [24]. Accordingly,
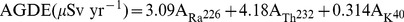
(4)


### Scanning Electron Microscope and Energy Dispersive X- ray Micro-analysis (SEM-EDAX)

The SEM-EDAX analyses were performed to investigate the morphological characteristics as well as for semi-quantifying the elemental composition. The sand samples were dried and subsequently smoothened using an air blower so as to make it suitable for quantitative determination. The samples were then mounted on stubs with conductive carbon tape coated with carbon, and examined at low vacuum mode at 20–30 kV, using a SEM EDAX JOEL-MODEL 6390 with Secondary - Electron Detector and with a counting time of 120–180 seconds. Microscopic study with EDAX analysis revealed the heavy mineral assemblage in percentage levels in the sand samples.

## Results and Discussion

The present study revealed that the sand samples collected from Chavara – Neendakara contain radioactive elements at remarkably high levels. Based on HPGe Detector Gamma spectrometric measurements, it could be inferred that elements such as Uranium (^238^U), Thorium (^232^Th) and Potassium (^40^K) are the major causative factors for the radioactivity thus conforming to the previous observations [20], [21], [25]. The study also indicates that the source of radioactivity in the sand samples of this HBRA could be the Cereium type of Monazite inasmuch as only Ce monazite contains Th along with cerium, lanthanum, praseodymium and neodymium which are common in other varieties.

### Radioactivity Concentrations

Data on the activity concentration of primordial radionuclides such as ^238^U, ^232^Th and ^40^K in sand samples (all values reported as Bq kg^−1^) collected from Chavara – Neendakara placer deposits are given in Table 1, and are represented as CH-1 to CH-19. The percentage variation of ^238^U, ^232^Th and ^40^K (existing among the 19 samples) was found to be <4%, <1%, and <5%, respectively. Out of the 22 km stretch of the coastal area featured in our present study in Chavara–Neendakara belt, the activity of ^238^U was found to be the highest (6023 Bq kg^−1^) at Puthenthura (8°57′46.46′′N, 76°31′47.93′′E), while the lowest activity (213 Bq kg^−1^) was recorded at Chavara (8°58′9.59′′N, 76°31′42.55′′E), beside Indian Rare Earth Limited (IRE) (Table 1). Likewise, the highest activity of ^232^Th (41280 Bq kg^−1^) has been recorded (as in the case of ^238^U) at Puthenthura (8°57′46.46′′N, 76°31′47.93′′E), the lowest activity of ^232^Th (1839 Bq kg^−1^) having been recorded at Chavara (8°58′11.25′′N, 76°31′41.65′′E). The highest ^40^K activity (2531 Bq kg^−1^) was observed at Chavara (8°58′16.00′′N, 76°31′39.98′′E, near St. Francis Assissi Church (Chavara–Karithura), while the lowest activity (222 Bq kg^−1^) was recorded (8°58′11.25′′N,76°31′41.65′′E) beside IRE in Chavara Table 1.

**Table 1:** Activities of ^238^U, ^232^Th, and ^40^K radionuclides from Chavara –Neendakara Coastal belt (HBRA).

No	Sample Code	SamplingSite	Geographical coordinates		Activity(Bq kg^−1^)			Absorbed Dose rate (D)(nGy h^−1^)	Observed by Scintillometer(nGy h^−1^)
			Latitude	Longitude	^238^ U	^232^Th	^40^ K		
**1**	CH-1	Chavara(Near St.Francis Assissi Church) Karithura	8°58′17.74′′N	76°31′39.45′′E	2929	13592	276	9717	9000
**2**	CH-2		8°58′16.00′′N	76°31′39.98′′E	5786	36229	2531	25114	18000
**3**	CH-3		8°58′15.05′′N	76°31′40.48′′E	3985	20862	1563	14745	16000
**4**	CH-4		8°58′14.82′′N	76°31′40.90′′E	3560	17999	1319	12772	12000
**5**	CH-5		8°58′13.70′′N	76°31′41.20′′E	2097	10037	628	7165	8000
**6**	CH-6		8°58′12.83′′N	76°31′41.41′′E	336	2071	274	1443	2000
**7**	CH-7	Chavara(Near IRE Building)	8°58′12.12′′N	76°31′41.62′′E	1930	15026	1385	10230	9000
**8**	CH-8		8°58′11.25′′N	76°31′41.65′′E	399	1839	222	1324	1000
**9**	CH-9		8°58′10.22′′N	76°31′41.82′′E	4448	29135	2230	20117	17000
**10**	CH-10		8°58′9.59′′N	76°31′42.55′′E	213	12013	1060	7609	8000
**11**	CH-11		8°58′6.98′′N	76°31′42.20′′E	2853	15324	454	10769	10000
**12**	CH-12	Puthenthura	8°57′57.63′′N	76°31′44.34′′E	1423	6629	915	4770	5000
**13**	CH-13		8°57′56.84′′N	76°31′44.63′′E	1365	6618	1040	4744	5000
**14**	CH-14		8°57′56.14′′N	76°31′44.99′′E	1854	9594	968	6801	8000
**15**	CH-15		8°57′47.45′′N	76°31′47.62′′E	1723	8319	1006	5953	5000
**16**	CH-16		8°57′46.46′′N	76°31′47.93′′E	6023	41280	2503	28356	21000
**17**	CH-17	Neendakara	8°57′45.77′′N	76°31′48.48′′E	639	3366	841	2403	3000
**18**	CH-18		8°56′53.26′′N	76°32′5.73′′E	1632	8480	995	6014	7000
**19**	CH-19		8°56′49.65′′N	76°32′6.63′′E	1560	7618	990	5447	7000
**20**	A-1 to A-10	Alleppey (Arabian Sea Coast)	9°29′28.06′′N	76°19′5.18′′E	37	56	BDL*	50	100
**21**	C-1 to C-10	Chennai (Bay of Bengal Coast)	13°3′18.55′′N	80°17′4.18′′E	BDL*	BDL*	446	19	100

* Below Detection Limit.

In Alleppey, a Normal Background Radiation Area (NBRA), the 10 sampling locations (A-1 to A-10) showed an average activity of 37.5 Bq kg^−1^ for ^238^U, while in the ten locations in Chennai (NBRA) (C-1 to C-10), the levels were less than 3.39 Bq kg^−1^, to be designated as below detection limit (BDL). For ^232^Th, the sand samples from Alleppey (NBRA) were showing only very little activity, while in Chennai (NBRA), the activity was below 6.64 Bq kg^−1^(BDL).

The rich presence of ^232^Th in the sand samples of the Chavara – Neendakara placer deposits had been attracting the attention of researchers for several years. There have been some attempts to quantitatively survey the radioactivity prevailing in this area [20], [21]. These surveys, however, have generated varying results, indicating that the amount of the radiation source in the sand sample could vary temporally and/or spatially. As per the previous assessments made by Shetty *et al*. 2010 [20], the activity of ^238^U ranged between 961 Bq kg^−1^ and 1116 Bq kg^−1^, while activities for ^232^Th and ^40^K have been found to vary from 6707 Bq kg^−1^ to 8099.6 Bq kg^−1^ and 162.64 Bq kg^−1^ to 2461 Bq kg^−1^ respectively. Our estimates (represented in Table 1), however, were observed to be higher than these values. It is difficult to ascertain the cause for the existence of such variations in activity of samples collected from same locality. Admittedly, variations are inevitable due to several factors such as the disturbances caused by human habitation.

The strategy of our present study also encompassed collection and testing the sand samples for (D) from all the 19 sites from the 22 km belt in Chavara-Neendakara HBRA. Out of these sites, the value of (D) was found to be at its lowest (1443 nGy yr^−1^) near St. Francis Assissi Church (Chavara-Karithura, 8°58′12.83′′N, 76°31′41.41′′E) while (D) was shown to be maximum at Puthenthura (8°57′46.46′′N, 76°31′47.93′′E) 28356 nGy h^−1^with an average value of 9763 nGy h^−1^ (Table 1). The average values of the (D) from the 10 sampling sites of NBRA, on the other hand, were found to be 50 nGy h^−1^ and 19 nGy h^−1^ in Alleppey and Chennai respectively as in Table 1.

Among the radionuclides detected from the HBRA, the highest activity was observed for ^232^Th (mean value 14000 Bq kg^−1^), while ^40^K (1116 Bq kg^−1^) showed the least activity Table 2. The data in (Table 1) also reveals that ^238^U and ^232^Th activities in Chavara – Neendakara (HBRA) soil samples are significantly higher than (P<0.001) those from Alleppey and Chennai (NBRA).

**Table 2:** Statistical data for radioactivity concentrations of ^238^U, ^232^Th and ^40^K (Bq kg^−1^) in the beach sand samples from the Chavara –Neendakara Placer deposits (HBRA).

	^238^U	^232^Th	^40^K
**Range**	1839–41280	213–6022	222–2531
**Arithmetic Mean(AM)**	2355	14000	1116
**Standard Deviation(SD)**	1726	11025	689
**Median**	1854	10037	1116
**Kurtosis**	0.9	1.2	0.3

**Figure 2:**
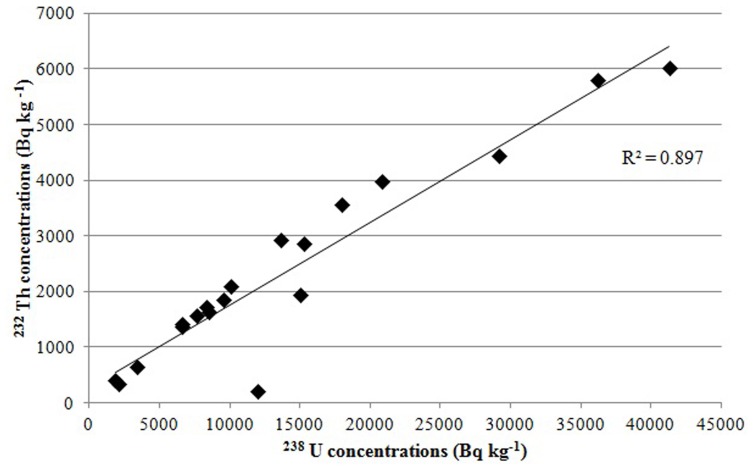
Correlation between ^232^Th and ^238^U in Chavara-Neendakara soils by gamma-ray spectrometry.

The present study reveals the occurrence of high positive correlation (r = 0.88, P<0.0001, Figure 2) between the distribution of ^238^U and ^232^Th, suggesting significant interdependence between the two variables. Pertinently enough, Nair *et al.* (2000) [25] in their extensive studies on the distribution of ^40^K,^238^U and ^232^Th in the soil of Karunagapally Taluk (including Chavara and Neendakara) had reported the existence of a positive correlation between the dose rate at 1 m above ground level and dose rate calculated from ^238^U, ^232^Th and ^40^K concentration in the soil. The existence of significant correlation between the ^238^U and ^232^Th content has been reported in other areas such as Jordan [27] Brazilian igneous rocks [28] and Eastern coast of Orissa [29].

### Dose Rate Measurements

An estimate of the annual external effective dose rates (AEED) depicts a measure of the effective dose equivalent to be received by the public due to soil radioactivity, as computed from the activity concentrations of ^232^Th, ^238^U and ^40^K in bulk sand samples from Chavara HBRA, and assessed by the Equation (3), as represented in Table 3. AEED in Chavara–Neendakara region varied from 1.19 mSv y^−1^ to 9.33 mSv y^−1 ^with an average of 1.2 mSv y^−1^. Reviewing the previous reports, it becomes obvious that the AEED values at Chavara – Neendakara are considerably higher than those of several other HBRAs and also the world average (0.07 mSv y^−1^). The values of AEED from Northern Jordan, for instance, was reported to be 63.2 u Sv y^−1^ [27] while that from Preta Beach of Brazil was reported to be 0.15 m Sv y^−1^ [30]. However, on the contrary, the AEED dose from Orissa (2.0 mSv y^−1^) was found to be higher than that of Chavara [29]. The radium equivalent activity, which is a widely used hazard index and a measure of the radiation dose likely to be delivered externally to the general public, was determined by assuming the gamma-ray dose rate produced by ^238^U, ^232^Th, and ^40^K as per Equation (1). Accordingly, the average (radium equivalent) activity in Chavara-Neendakara was found to be 23174 Bq kg^−1^, higher than highlands of Jordan [27].

**Table 3:** Average values of Radium equivalent activity (Ra_eq_), Annual effective dose equivalent rates (AEED) and Annual gonadal dose equivalent (AGDE) in soil samples collected from the studied areas.

Province	Ra eq(Bq kg^−1^)	AEED(µSv yr^−1^)	ADGE(mSv yr^−1^)
**HBRA**(Chavara-Neendakara)(Arabian Sea Coast)	23174.2	8.79	66.15
**NBRA**(Alleppey)(Arabian Sea Coast)	117.7	0.64	0.35
**NBRA**(Chennai)(Bay of Bengal Coast)	34.451	0.22	0.14

AGDE computed from the specific activities of ^226^Ra, ^232^Th and ^40^K (Equation 4) was found to be 66.12 mSv y^−1^ in Chavara –Neendakara and 0.350 mSv y^−1^ in Alleppey and 0.140 mSv y^−1^ in Chennai Table 3. The AGDE value reported in our present study is found to be higher than what was reported from other places such as highlands of Jordan 0.333 mSv yr^− 1^ [27] and Egypt 0.12 mSv y^−1^ [21], the threshold limits recommended by ICRP being 1.0 mSv y^−1^ [31]. The average absorbed dose rates (D) (9795 nGy h^−1^) computed from the present study is found comparable with the top-ranking HBRAs in the world, thus offering the Chavara –Neendakara placer the second position, after Brazil (Table 4). It is also pertinent that this value (equivalent to: 85.8 mGy^−1^) is much higher than the World average of 0.5 mGy y^−1^ [12]. This perceptibly high absorbed gamma dose rate (D), entrained with the high AEDE and AGDE values Table 3 existing in this HBRA warrants for a precise assessment of the impact of the background radiation, if any, on the organisms that inhabit along this placer deposit. National Research Council report on Health Effects of Low Levels of Ionizing Radiation reveals the occurrence of an increased frequency of chromosome aberration in areas of high natural background radiation [32]. The increases are very consistent with radiation workers and in persons exposed at high dose levels. No increase in the frequency of cancer was, however, documented in populations residing in areas of high natural background radiation. Likewise, the cytogenetic studies conducted on newborns and adults, reported the occurrence of chromosomal abnormalities [33], [34] although these abnormalities were not different from those of the normal background radiation areas. Furthermore, the studies on telomere length among the adult inhabitants also revealed no significant abnormality [18]. Quite recently, the studies on telomere length among the adult inhabitants revealed no significant abnormality [18].

**Table 4:** Comparison of radiation dose rates of Chavara-Neendakara beach placer deposit area with other HBRAs.

S.No	Area (Country)	Characteristics of Area	Absorbed dose rate in air	Reference
			(nGy h^−1^)	
	**Present Study**			
**1**	Chavara Neendakara (India)	Monazite sands	1475–28,388	–
**2**	Guarapari (Brazil)	Monazite sands	90–90,000	[40]
**3**	Ramsar (Iran)	226Ra in spring water	70–17,000	[12]
**4**	Southwest (France)	Uranium minerals	10–10,000	[13]
**5**	Morro Do Forro (Brazil)	Thorium rich alkaline intrusive	2800	[30]
**6**	Nile Delta (Egypt)	Monazite sands	20–400	[31]
**7**	Cox’s Bazar (Bangladesh)	Monazite sands	260–400	[44]
	**India**			
**8**	Kerala coast	Monazite sands	200–4000	[10]
**9**	Ullal (Karnataka)	Monazite sands	2100	[14]
**10**	Tamil Nadu coast	Monazite sands	200–4000	[11]
**11**	Kudiraimozhi (Tamil Nadu)	Monazite sands	200–900	[43]
**12**	Bhimilipatanam (Andhra Pradesh)	Monazite sands	200–3000	[43]
**13**	Kalpakkam (Tamil Nadu)	Monazite sands	3500	[15]
**14**	Chhatrapur (Orissa)	Monazite sands	375–5000	[29]

### SEM-EDAX Analysis

**Figure 3:**
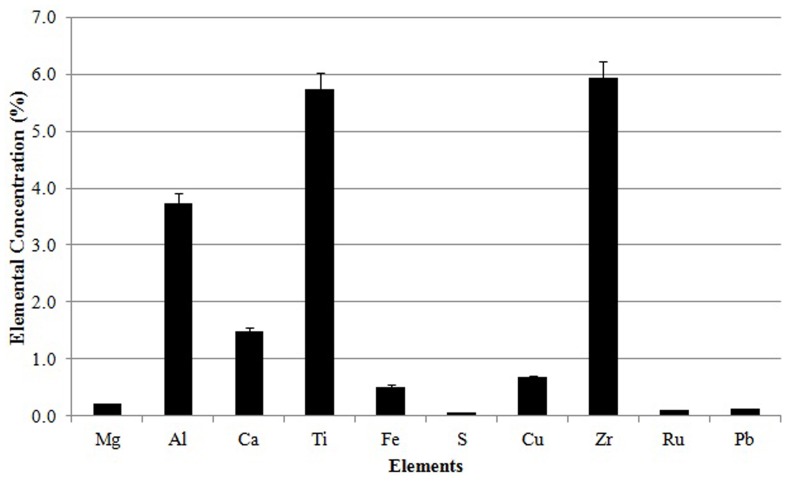
Elemental concentration in the Chavara-Neendakara placer deposits by SEM-EDAX analysis.

The present SEM-EDAX analyses reveal that titanium (Ti) and zircon are the major trace elements in the sand samples; the distribution of Ti ranged from 0.76% to 21.73% with a mean value of 5.7% while that of zircon (Zr) ranged between 0.91% and 16.13% with an average of 5.9%. The other trace elements observed were aluminum (mean = 3.7%), copper (mean = 0.7%), iron (mean = 0.5%), ruthenium (mean = 0.1%), magnesium (mean = 0.2%), calcium (mean = 1.5%), sulphur (mean = 0.1%) and lead (mean = 0.1%) Figure 3. We are further encouraged to collate the results of the study performed by Lal *et al.* (1989) [35] on the elemental analysis of Indian monazite ore, wherein the authors have reported the occurrence of zircon and lead, in addition to U and Th. We were also able to compare our data (on trace elements) with those of the Egyptian sand samples. It reveals that Ti and Zr, the major components of the sand samples of Chavara – Neendakara, were not present in significant levels in the Egyptian sample [36].

Detailed gamma spectrometric analysis of the results clearly reveals that the high background radiation existing in Chavara Neendakara belt is caused by the presence of ^232^Th and ^238^U to a considerable extent, and ^40^K to a limited extent. The study further indicates that the radionuclide (^232^Th, ^238^U and ^40^K) concentration is not uniformly distributed all the way through the 22 km stretch of the HBRA; results of our sampling indicate that there exists a space – dependent variation of radioactivity along the entire coastal stretch of the HBRA (Chavara-Neendakara). The strong positive correlation (r = 0.88; P<0.005) existing between the ^238^U and ^232^Th concentrations in this HBRA indicates that monazite could be the major source of the background radiation prevalent in this area, and that the profiles of these two elements are interdependent. The study has also helped identification of other trace elements present in Chavara-Neendakara. The average absorbed dose rates (D) (9795 nGy h^−1^) computed from the present study is found to be comparable with the top-ranking HBRAs in the world, thus offering the Chavara-Neendakara placer the second position, after Brazil. It would be worth recalling at this juncture, some of the investigations performed to assess the impact of human exposure to the background radiation in this region. Nair *et al.* (2009) [37], for instance, in their attempt to evaluate the health effects of high background radiation (HBR) on human population, had made an extensive study involving approx 4,00,000 individuals living in 12 Panchayats of Karunagapalli Taluk, including Chavara and Neendakara. Their studies have categorically proved that there exists no correlation between human exposure to high-level natural radiation and cancer risk. Other abnormalities such as abortion, Down’s syndrome, congenital malformation and/or chromosomal aberrations were also found to have no correlation with exposure to HBR [18], [33], [34], [38] Forster *et al*. (2002) [39] on the other hand, reported an association of the exposure to natural radioactivity with the occurrence of mitochondrial DNA mutations in the human inhabitants, evincing abnormalities at the genotype levels. Obviously, more research is warranted before making any generalizations on the impact of background radiation on the human system at large. And virtually no study exists on the lower organisms that inhabit this area and may have a tolerance limit much less than the humans. Pertinently, the lower organisms may enter the food chain and subsequently result in radioactive accumulation in the human inhabitants. In this context, it would be worth mentioning about the sabellarid sand castle worms (*Phragmatopoma* sp.) inhabiting along the Chavara-Neendakara coast.

These polychaete (Annelid) worms are seen dwelling within small tube-like enclosures formed out of agglutinated sand; the glue-like substance for the agglutination is suggested to be the secretory product of the epithelium. Interestingly, our HPGe (gamma spectrometer) analysis revealed that this agglutinated sand that affords a crest-like protection, is in close proximity to the body of the organism itself, and consists of ^238^U (3212 Bq kg^−1^), ^232^Th (15865 Bq kg^−1^) and ^40^K (2018 Bq kg^−1^), comparable to those of the sand samples from HBRA (Derin *et al.* unpublished observations). Further, we could also find the presence of several other organisms, primarily invertebrates (edible arthropods and mollusks, for instance) that inhabit along this placer deposit. Future research could address the issue of the possible impact of natural radiation on the biota inhabiting this HBRA.

## References

[1] UNSCEAR (2000) Sources and effects of ionizing radiation. United Nations Scientific Committee on the Effect of Atomic Radiation, United Nations, New York. 10.1088/0952-4746/21/1/609 11281539

[2] UNSCEAR (1993) Ionizing Radiation Sources and Effects on Ionizing Radiation. United Nations Scientific Committee on the Effects of Atomic Radiation, United Nations, New York.

[3] Paschoa AS (2000) More than forty years of studies of natural radioactivity in Brazil. Technol 7: 193–212.

[4] Bennett BG (1997) Exposure to natural radiation worldwide. In: Proceedings of the Fourth International Conference on High Levels of Natural Radiation: Radiation Doses and Health Effects. Beijing, China. Elsevier, Tokyo. 15–23.

[5] Wei L, Sugahara T (2000) An introductory overview of the epidemiological study on the population at the high background radiation areas in Yangjiang, China. J Radiat Res 41: 1–7. 10.1269/jrr.41.s1 11142208

[6] Wei L, Zha Y, Tao Z (1993) Epidemiological investigation in high background radiation areas in Yang jiang, China. In: Proceedings of the International Conference on High Levels of Natural Radiation. Ramsar, IAEA, Vienna. 523–547.

[7] Paul AC, Pillai PMB, Haridasan P, Radhakrishnan S, Krishnamony S (1998) Population exposure to airborne thorium at the high natural radiation areas in India. J Environ Radioact 40: 251–259.

[8] Mishra UC (1993) Exposure due to the high natural radiation background and radioactive springs around the world. In: Proceedings of the International Conference on High Level Natural Radiation Areas. Ramsar, Iran. IAEA Publication Series, IAEA, Vienna. 29.

[9] Sunta CM (1993) A review of the studies of high background radiation areas of the S-W coast of India. In: Proceedings of the International Conference on High levels of Natural Radiation Areas. Ramsar, Iran. IAEA Publication Series, IAEA, Vienna. 71–86.

[10] Sunta CM, David M, Abani MC, Basu AS, Nambi KSV (1982) Analysis of dosimetry data of high natural radioactivity areas of southwest coast of India. In: The Natural Radiation Environment. Wiley Eastern Limited, India. 35–42.

[11] Sohrabi M (1998) The state-of-the-art on worldwide studies in some environments with elevated naturally occurring radioactive materials (NORM). Appl Radiat Isot 49: 169–188. 9451771 10.1016/s0969-8043(97)00238-8

[12] Ghiassi-nejad M, Mortazavi SMJ, Cameron R, Niroomand-rad A, Karam PA (2002) Very high background radiation areas of Ramsar, Iran: preliminary biological studies. Health Phys 82: 87–93. 11769138 10.1097/00004032-200201000-00011

[13] Delpoux MA, Dulieu LA, Dalebroux M (1997) Experimental study on the genetic effects of high levels of natural radiation in south France. In: Proceedings of the Fourth International Conference on High Levels of Natural Radiation: Radiation Doses and Health Effects, 1996, Beijing, China. Elsevier, Tokyo. 397–406.

[14] Radhakrishna AP, Somasekarapa HM, Narayana Y, Siddappa K (1993) A new natural background radiation area on the southwest coast of India. Health Phys 65: 390–395. 8376119 10.1097/00004032-199310000-00006

[15] Kannan V, Rajan MP, Iyengar MAR, Ramesh R (2003) Distribution of natural and anthropogenic radionuclides in soil and beach sand samples of Kalpakkam (India) using hyper pure germanium (HPGe) gamma ray spectrometry. Appl Radiat Isot. 57: 109–119. 10.1016/s0969-8043(01)00262-7 12137019

[16] Alam MN, Chowdhury MI, Kamal M, Ghose S, Islam MN, Mustafa MN, et al (1999) The ^226^Ra, ^232^Th and ^40^K activities in beach sand mineral and beach soils of Cox’s Bazar, Bangladesh. J Environ Radioact 46: 243–250.

[17] Kochupillai N, Verma IC, Grewal MS (1976) Down’s Syndrome and related abnormalities in an area of high background radiation in coastal Kerala. Nature 272: 60–61. 10.1038/262060a0 132614

[18] Das B (2010) Genetic studies on human population residing in High level natural radiation areas of Kerala coast. In: BARC News letter. 28–37.

[19] Nair KRR, Nair MK, Gangadharan P, Jayadevan S, Jayalakshmy P (2000) Natural Background Radiation Level Measurement in Karunagappally taluk, Kerala,India. In: Proceedings of the 5th International Conference on High Levels of Natural Radiation & Radon Areas: Munich, Germany. 79–82.

[20] Shetty PK, Narayana Y (2010) Variation of radiation level and radionuclide enrichment in high background area. J Environ Radioact 30: 1–5. 10.1016/j.jenvrad.2010.08.003 20833457

[21] Narayana Y, Shetty PK, Siddappa K (2005) Enrichment of Natural radionuclides in monazite areas of coastal Kerala. Intr Congr Ser 1276: 333–334. 10.1016/j.jenvrad.2005.08.002 16213068

[22] EML Procedures Manual (1983) In:26th edn. Environmental Measurement Laboratory, US Department of Energy, NewYork.

[23] Senthilkumar B, Dhavamani V, Ramkumar S, Philominathan P (2009) measurement of gamma radiation levels in soil samples from Thanjavur using gamma ray spectrometry and estimation of population exposure. J Med Phys 35(1): 48–53. 10.4103/0971-6203.55966 PMC2825004 20177570

[24] Mamont-Ciesla K, Gwiazdowski B, Biernacka M, Zak A (1982) Radioactivity of building materials in Poland. In: Natural Radiation Environment. Newyork: Halsted Press. 551 p.

[25] Nair KRR, Nair MK, Gangadharan P, Jayalakshmy P, Jayadevan S (2000) Distribution of ^238^U, ^232^Th and ^40^K in the soils of Karunagappally Taluk - A high background radiation area in India. In: Proceedings of the 5th International Conference on High Levels of Natural Radiation & Radon Areas: Munich, Germany. 404–407.

[26] IAEA (1989) Construction and Use of Calibration Facilities for Radiometric Field Equipment Technical Report 309, Vienna, Austria.

[27] Ibrahim AF, Mohammad AI (2009) Soil radioactivity levels and radiation hazard assessment in the highlands of northern Jordan. Radiat Meas 44: 102–110.

[28] Moura CL, Artur AC, Bonotto DM, Guedes S, Martinelli C (2011) Natural radioactivity and radon exhalation rate in Brazilian igneous rocks. Appl Radiat Isot 69: 1094–1099. 21459585 10.1016/j.apradiso.2011.03.004

[29] Mohanty AK, Sengupta D, Das SK, Vijayan V, Saha SK (2004) Natural radioactivity in the newly discovered high background radiation area on the eastern coast of Orissa, India. Radiat Meas 38: 153–165.

[30] Freitas AC, Alencar AS (2004) Gamma dose rates and distribution of natural radionuclides in sand beaches-Ilha Grande, Southeastern Brazil. J Environ Radioact 75: 211–223. 15172728 10.1016/j.jenvrad.2004.01.002

[31] ICRP 60 (1990) Recommendations of the International Commission on Radiological Protection. ICRP Publication 60. In: Annals of the ICRP. Oxford UK: Pergamon Press. 21: 1–3. 2053748

[32] BEIR V (1990) Health Effects of Exposure to Low Levels of Ionizing Radiation Committee on the Biological Effects of lonizing Radiation. European Nuclear Society, Belgium.

[33] Thampi MV, Cheriyan VD, Kurien CJ, Ramachandran EN, Karuppasamy CV (2002) Cytogenetic studies in the high level natural radiation areas of Kerala High Levels of Natural Radiation and Radon areas. Intr Congr Ser 1225: 207–211.

[34] Cheriyan VD, Kurien CJ, Das B, Ramachandran EN, Karuppasamy CV, et al (1999) Genetic monitoring of the human population from high level natural radiation areas of Kerala on the south-west coast of India. II Incidence of numerical structural and chromosomal aberrations in the lymphocytes of newborns. J Radiat Res 152: 154–158. 10564959

[35] Lal M, Choudhury RK, Joseph D, Bajpai HN, Tyer CSP (1989) Elemental analysis of selected Indian monazite ores by energy dispersive X-ray fluorescence/EDXRF/spectroscopy. J Radioanal Nucl Chem Letters 137/2: 127–133.

[36] Hassan AM, Abdel-Wahab M, Nada A, Walley El-Dine N, Khazbak A (1997) Determination of uranium and thorium in Egyptian monazite by gamma-ray spectrometry. Appl Radiat Isot 48: 149–152.

[37] Nair KRR, Rajan B, Akiba S, Jayalekshmi P, Nair MK, Gangadharan P, Koga T, Morishima H, Nakamura S, Sugahara T (2009) Background radiation and cancer incidence in Kerala, India–Karunagappally cohort study. Health Phys 96(1): 55–66. 19066487 10.1097/01.HP.0000327646.54923.11

[38] Jaikrishan G, Andrews VJ, Thampi MV, Koya PK, Rajan VK, Chauhan PS (1999) Genetic monitoring of the human population from high-level natural radiation areas of Kerala on the southwest coast of India. I. Prevalence of congenital malformations in newborns. Radiat Res 152: 149–153. 10564958

[39] Forster L, Forster P, Lutz-Bonengel S, Willkomm H, Brinkmann B (2002) Natural radioactivity and human mitochondrial DNA mutations. In: Proceedings National Academy of Sciences USA (99) 13950–13954. 10.1073/pnas.202400499 PMC129803 12370437

[40] Pfeier WC, Penna-Franca E, Costa Ribeiro C (1981) Measurements of environmental radiation exposure dose rates at selected sites in Brazil. An Acad Bras Cienc 53: 683–691. 7345962

[41] Veiga L, Amaral E, Magalhaes M (1999) Brazilian areas of elevated levels of natural radiation: a critical review and relevant future studies. In: Second Symposium on Technologically Enhanced Natural radiation, Rio de Janerio, Brazil.

[42] El-Khatib AM, El-Kher AA (1988) Regional study of black sands radioactivity. Isotopenpraxis 24: 333–336.

[43] Paul AC, Pillai PMB, Haridasan P, Radhakrishnan S, Krishnamony S (1998) Population exposure to airborne thorium at the high natural radiation areas in India. J Environ Radioact 40: 251–259.

[44] Mollah AS, Das SC, Begum A, Rahman MM, Molla MAR (1989) Indoor gamma radiation exposure at the Cox’s Bazar coastal areas. Radiat Prot Dosim 27: 43–45.

